# Hybrid-FHR: a multi-modal AI approach for automated fetal acidosis diagnosis

**DOI:** 10.1186/s12911-024-02423-4

**Published:** 2024-01-22

**Authors:** Zhidong Zhao, Jiawei Zhu, Pengfei Jiao, Jinpeng Wang, Xiaohong Zhang, Xinmiao Lu, Yefei Zhang

**Affiliations:** 1 School of Cyberspace, Hangzhou Dianzi University, Hangzhou, China; 2 College of Electronics and Information Engineering, Hangzhou Dianzi University, Hangzhou, China

**Keywords:** Fetal heart rate, Fetal acidosis, Cardiotocography, Cross-modal feature fusion, Multi-modal, Temporal convolutional network, Attention mechanisms

## Abstract

**Background:**

In clinical medicine, fetal heart rate (FHR) monitoring using cardiotocography (CTG) is one of the most commonly used methods for assessing fetal acidosis. However, as the visual interpretation of CTG depends on the subjective judgment of the clinician, this has led to high inter-observer and intra-observer variability, making it necessary to introduce automated diagnostic techniques.

**Methods:**

In this study, we propose a computer-aided diagnostic algorithm (Hybrid-FHR) for fetal acidosis to assist physicians in making objective decisions and taking timely interventions. Hybrid-FHR uses multi-modal features, including one-dimensional FHR signals and three types of expert features designed based on prior knowledge (morphological time domain, frequency domain, and nonlinear). To extract the spatiotemporal feature representation of one-dimensional FHR signals, we designed a multi-scale squeeze and excitation temporal convolutional network (SE-TCN) backbone model based on dilated causal convolution, which can effectively capture the long-term dependence of FHR signals by expanding the receptive field of each layer’s convolution kernel while maintaining a relatively small parameter size. In addition, we proposed a cross-modal feature fusion (CMFF) method that uses multi-head attention mechanisms to explore the relationships between different modalities, obtaining more informative feature representations and improving diagnostic accuracy.

**Results:**

Our ablation experiments show that the Hybrid-FHR outperforms traditional previous methods, with average accuracy, specificity, sensitivity, precision, and F1 score of 96.8, 97.5, 96, 97.5, and 96.7%, respectively.

**Conclusions:**

Our algorithm enables automated CTG analysis, assisting healthcare professionals in the early identification of fetal acidosis and the prompt implementation of interventions.

**Supplementary Information:**

The online version contains supplementary material available at 10.1186/s12911-024-02423-4.

## Background

Fetal acidosis is an imbalance in the acid-base balance of the fetus’s body that causes the fetus’s blood to become too acidic [1]. Fetal acidosis caused by hypoxia can lead to multiple organ damage, and even death. Therefore, we need a safe and effective method for early detection of fetal acidosis to assist obstetricians in determining whether intervention measures during childbirth are required.

Cardiotocography (CTG), also known as electronic fetal monitoring (EFM), is a common monitoring technique wherein clinicians assess the fetal health by analyzing signals related to the Fetal Heart Rate (FHR) and uterine contractions (UC) obtained from CTG. While CTG has become the most widely employed fetal monitoring method [2], its utility remains a subject of debate due to high interobserver (different specialists at the same time) and intraobserver (same specialist at different times) variability. Furthermore, CTG may lead to an increase in false positives and a higher rate of planned deliveries [3, 4]. Consequently, there is an urgent need to develop an automated diagnostic technique to address these limitations.

Previously, researchers employed morphological time domain, frequency domain, and nonlinear domain parameters of FHR signals for feature extraction, feature selection, and classification. Georgieva et al. [5] extracted 12 clinical parameter features, and researchers obtained a sensitivity of 60.3% and a specificity of 67.5% using a feedforward artificial neural network (ANN). Spilka et al. [6, 7] extracted a total of more than 50 features including the above three domain features, and used the Adaptive Boosting (AdaBoost) classifier and Random Forest classifier, respectively. Cömert et al. [8] used the short-time Fourier transform (STFT) and gray level co-occurrence matrix (GLCM) to extract the image-based time-frequency features (IBTF) from the FHR signal. Zhao et al. [9] extracted 47 expert features from FHR signals and utilized statistical testing (ST) and PCA for dimensionality reduction. Pini et al. [10] extracted 23 expert features and applied the recursive feature elimination (RFE) method to select the most relevant subset of features. These methods rely on expert features. Although they are highly reliable and interpretable, feature extraction can be complex and limited by the quality of the signal and domain-specific knowledge.

In the past decade, with the development of deep learning (DL), numerous studies demonstrated that deep neural networks have a wide range of applications in healthcare [11, 12]. Compared to traditional machine learning (ML) methods, these algorithms can learn important features automatically from the original input signal. This self-learning ability allows them to discover complex patterns in time series signals without the need for human feature engineering. Bursa et al. [13] and Cömert et al. [14] conducted research on two-dimensional convolutional neural network (2D-CNN) models. Bursa et al. utilized Continuous Wavelet Transform (CWT) on 1-dimensional fetal heart rate signals and contraction signals, and authors achieved a high classification accuracy of 94.1%. Cömert et al. used the STFT with transfer learning to analyze FHR signals. Li et al. [15] used a one-dimensional convolutional neural network (1D-CNN) and compared it with traditional feature extraction methods, demonstrating that 1D-CNN outperforms traditional methods. Liang et al. [16] proposed a one-dimensional convolutional neural network - gated recurrent unit (1D-CNN-GRU) model, and authors obtained an accuracy of 95.15%. Fei et al. [17] integrated three signals - FHR, UC, and fetal movement (FetMov) - by using an embedding layer to combine the features at the input level. Spairani et al. [18] proposed a hybrid method based on neural structures, where they converted FHR signals into the image domain, and researchers then parallelly input a set of expert features and finally perform decision fusion at the classification level.

However, most existing studies in FHR signal analysis are based on a single modality feature, which may not provide sufficient information to fully describe and analyze complex FHR signals. Moreover, FHR signals are often subject to various types of noise and interference, making single-modal features less stable and reliable. In contrast, multimodal features can capture a richer representation of potential features, and different modalities may have varying importance in different scenarios. By fusing multimodal features, the weights of each modality can be learned adaptively, thereby improving the accuracy of diagnosis.

**Fig. 1:**
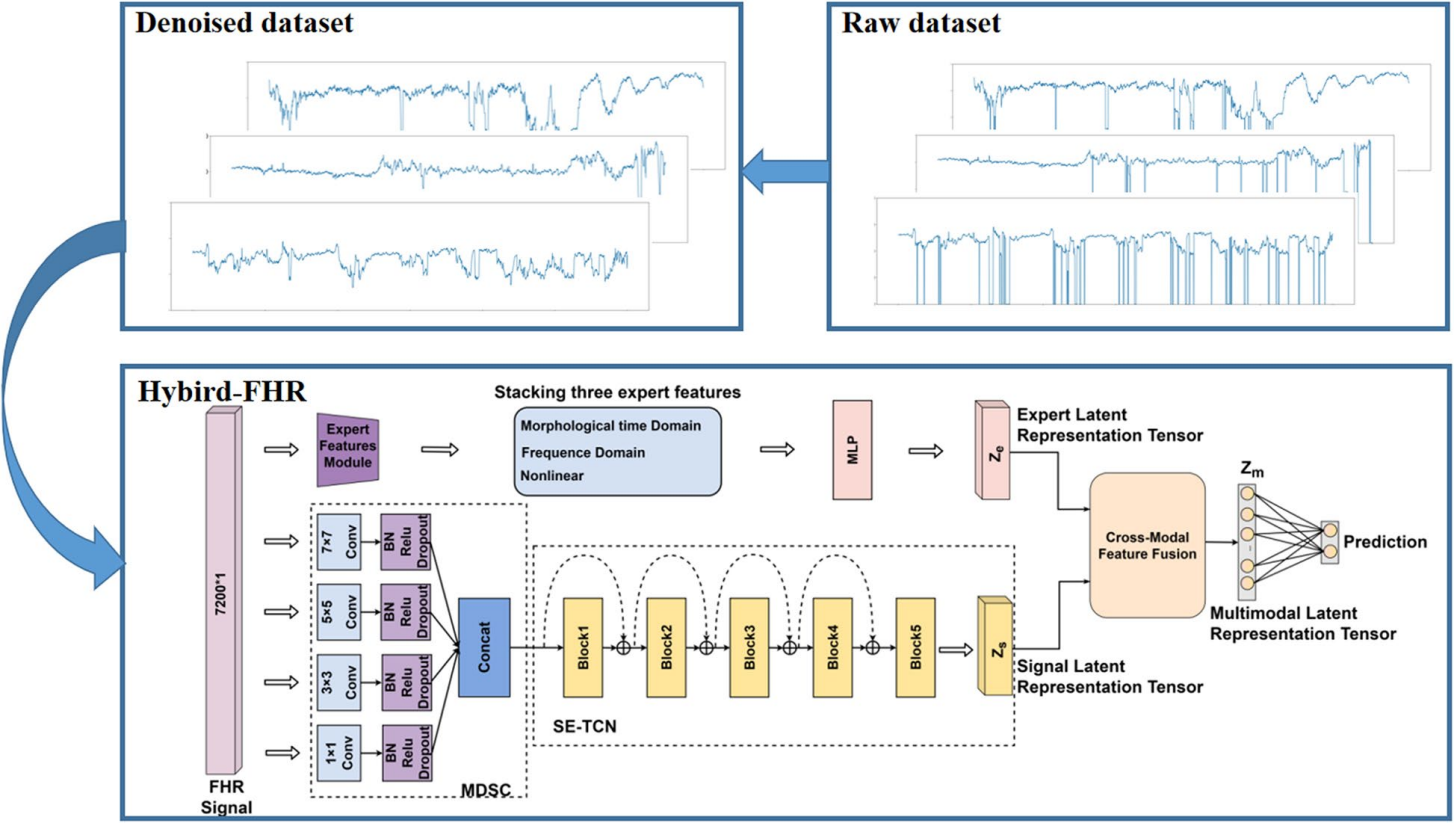
Overview of proposed method (Hybrid-FHR)

Based on the analysis presented, we propose a novel framework called Hybrid-FHR to diagnose fetal acidosis, assist doctors in identifying pathological fetuses, and reduce the rate of stillbirths. The algorithm utilizes multimodal features and combines the advantages of deep learning with expert prior knowledge. The overall framework of the Hybrid-FHR algorithm is depicted in Fig. 1.

The contributions and innovations of this study are listed as follows:

1. Our proposed fetal acidosis diagnostic framework (Hybrid-FHR) incorporates multimodal features and effectively leverages the information provided by various features. Through our experiments, we have demonstrated that our approach achieves significant performance gains in the diagnosis of fetal acidosis.
2. We designed a lightweight backbone network SE-TCN for extracting spatio-temporal representations of FHR signals, which utilizes dilated casual convolutions to effectively enhance the global perception capability of the entire network. Furthermore, a cross-modal feature fusion (CMFF) method based on multi-head attention mechanism is proposed to achieve optimal weighted fusion of different modalities.
3. We designed three types of expert features (morphological time domain, frequency domain, and nonlinear) by incorporating expert prior knowledge, which further improved the performance of the model.

## Methods

In this section, we first introduce the three types of expert features based on prior knowledge. Next, we elaborate on the SE-TCN backbone network for extracting features from one-dimensional FHR signals, and finally, we introduce the cross-modal feature fusion (CMFF) approach, which uses a multi-head attention mechanism to adaptively weight different modal features.

### Expert features module

Based on expert prior knowledge, we carefully designed a set of 45 features from the pre-processed FHR signals, including 21 morphological time domain, 14 frequency domain, and 10 nonlinear features. The specific formulas and details of these parameters can be found in (Additional files 1, 2, 3). These 45 features were processed through two layers of linear projection to obtain the expert latent representation tensor, denoted as *Z*_e_.

A. Morphological time domain

In this study, we calculated several morphological time domain characteristics following the International Federation of Gynecology and Obstetrics (FIGO) guidelines [19], including baseline (BL), number of accelerations (nACC), and number of decelerations (nDEC).

Time domain characteristics are mainly derived from the fetal heart rate variability (FHRV), which is the variability of the heartbeat cycle variation. To analyze the HRV, we must convert the FHR to RR (heartbeat-by-heartbeat) interval sequences with the following conversion equation:

1 $$RR=\frac{60000}{FHR}$$

The time difference between two consecutive RR intervals is called NN, which is calculated as follows:

2 $$NN= diff(RR)$$

In this study, we have referred to commonly used parameters for adult HRV and calculated various statistical measures to analyze the fetal heart rate variability signal in the time domain. These measures include basic parameters such as the maximum, minimum, mean, median, standard deviation, kurtosis, and skewness of the RR interval. Other parameters include the standard deviation of NN (SDNN), the root mean square of successive differences of RR intervals (RMSSD), NN50, and pNN50, which determine the number and percentage of NN that differ by more than 50 ms. Short-term variability and long-term variability (STV and LTV [20]), the triangular index (Tri [21]), and the triangular interpolation of the NN interval histogram (TINN [22]) were also calculated.

The morphological time domain features of the FHR are therefore as follows:

- Morphological time domain: {mean_baseline, max_baseline, min_baseline, std_baseline, nACC, nDEC, max_rr, min_rr, mean_rr, median_rr, std_rr, skew_rr, kurt_rr, SDNN, RMSSD, NN50, pNN50, STV, LTV, Tri, TINN}.

B. Frequency domain

The spectral analysis of FHRV examines changes in the fetal autonomic nervous system (ANS) activity [23], which can be observed in the periodic changes in FHRV. We followed the suggestion in [24] to divide the frequency range into four bands: very low frequency (VLF, 0–0.03 Hz), low frequency (LF, 0.03–0.15 Hz), medium frequency (MF, 0.15–0.5 Hz), and high frequency (HF, 0.5–1 Hz).

We used the Fast Fourier Transform (FFT) to convert the signal into the frequency domain and divided it into four frequency bands. We extracted the power spectral density, power spectral ratio, peak frequency, and total power spectral density of each frequency band. We also calculated the LF/(MF + HF) energy ratio. Therefore, the frequency domain features of FHR are as follows:

Frequence Domain: {rr_VLF, rr_LF, rr_MF, rr_HF, rr_Total_Power, rr_percent_VLF, rr_percent_LF, rr_percent_MF, rr_percent_HF, rr_peak_VLF, rr_peak_LF, rr_peak_MF, rr_peak_HF, rr_ratio,}.

C. Nonlinear

In recent years, nonlinear measurements for studying FHR kinetics have become increasingly available and have shown promising results [25–27]. We perform nonlinear feature extraction using the NeuroKit2 library in Python. The nonlinear methods used in this study include Poincare plot parameters [28], approximate entropy (ApEn, [29]), sample entropy (SampEn, [29]), Shannon entropy (ShannEn, [30]), fuzzy entropy (FuzzyEn, [29]), Lempel-Ziv complexity index (LZC, [31]), fractal dimension (FD, [32]), and Hurst index (Hurst, [33]), as follows:

- Nonlinear: {SD1, SD2, SD_Ration, ApEn, SampEn, ShannEn, FuzzyEn, LZC, FD, Hurst}.

Where SD1 and SD2 represent the short-axis and long-axis deviations of the Poincare plot, respectively, and SD_Ratio represents the ratio of the two.

### Signal backbone

This paper proposes a SE-TCN backbone network to extract latent feature representations of FHR signals. The network comprises a Multi-scale Depthwise Separable Convolution (MDSC) module and five SE-TCNBlocks. Table 1 presents the detailed hyperparameter settings and output dimensions of each layer of the proposed signal backbone.

**Table 1:** The detailed hyperparameter settings and output dimensions of each layer of the SE-TCN

Layer	Output shape	Kernel size	Stride	Padding	Dilation factor	Activation
Input	B × 7200 × 1	–		–	–	–
MDSC	B × 7200 × 64	[1, 3, 5, 7]	1	Same	1	ReLu
SE-TCNBlock1	B × 2400 × 64	15	3	Casual	2	ReLu
SE-TCNBlock2	B × 2400 × 64	15	1	Casual	4	ReLu
SE-TCNBlock3	B × 800 × 128	15	3	Casual	8	ReLu
SE-TCNBlock4	B × 800 × 128	15	1	Casual	16	ReLu
SE-TCNBlock5	B × 400 × 256	15	2	Casual	32	ReLu

*B* Batchsize

A. MDSC

Assuming that *X*_*s*_ ∈ ℝ^*B* × *N* × *C*^ is a whole representation of a set of continuous one-dimensional FHR signals, where subscript *s* is an abbreviation for signal, and ℝ denotes the real numbers set, B represents the batchsize, N represents the signal length, and C represents the number of channels.

Before the FHR signal passes through the SE-TCNBlocks, we designed a MDSC module for capturing signal features at different scales. In MDSC, we adopt depthwise separable convolution (DSC [34]) to replace ordinary convolution. DSC decomposes the convolution operation into depthwise convolution and pointwise convolution. The former performs convolution only on each input channel, while the latter performs convolution on the output channels. Compared to ordinary convolution, DSC can effectively reduce the number of parameters and computation, thereby improving model efficiency.

MDSC combines multiple DSCs of different scales. Different-sized convolution kernels move along the one-dimensional direction to extract features from the entire signal, gradually obtaining features that can fully represent the sequence in a locally-aware manner. The four different channels of convolution kernels in MDSC have sizes of 1, 3, 5, and 7, with dilation factors of 1 and channel numbers of 16. Finally, by fusing the outputs of different convolution kernel channels, a tensor with a channel number of 64 is obtained.

B. SE-TCNBlock

The 1D convolution method is often used for feature extraction in time series data. For long-series problems such as FHR signals, the normal convolutional approach (dilation factors *d* = 1) is prone to phenomena such as gradient disappearance, which is not satisfactory. To increase the long time dependence of the network and to improve its ability to reach into the past for prediction, a temporal convolutional network (TCN) was proposed [35, 36].

**Fig. 2:**
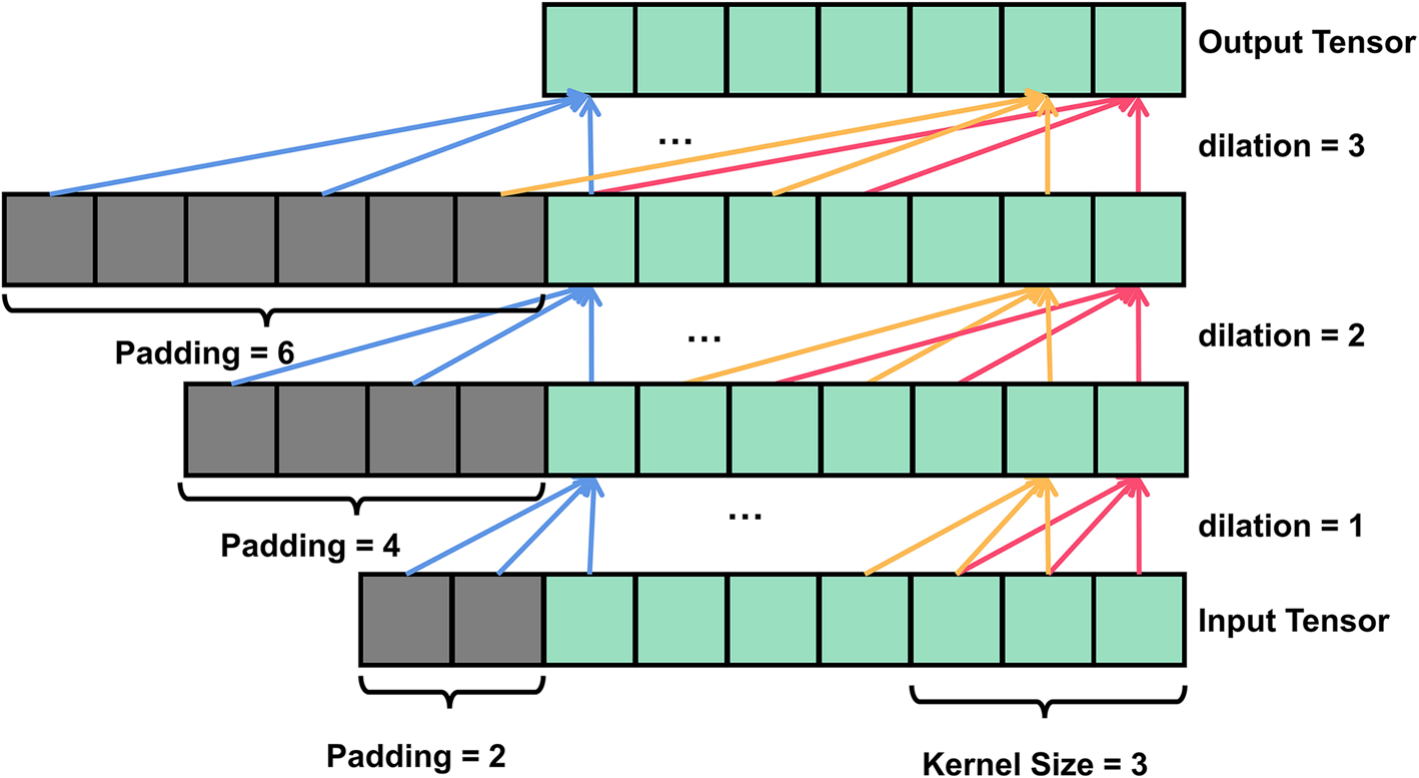
A Dilated Causal Convolution with dilation factors d = 1, 2, 3 and kernel size k = 3

TCN combines causal with dilated convolution, and Fig. 2 depicts the dilated causal convolution with dilation factors *d* = 1, 2, 3 and a convolution kernel size *k* = 3. The output at a certain moment is only related to the current and the past moments, using a zero-padding approach with the number of paddings per layer equal to *d* × (*k* ‐ 1). Furthermore, the receptive field size (RFS) of the network increases exponentially with the number of layers. For a one-dimensional time series *X* and a convolution kernel *w* of size *k*, the dilated convolution can be expressed as follows:

3 $$Y(t)=\left(X{\ast}_dw\right)(t)=\sum \limits_{i=0}^{k-1}w(i)\cdot X\left(t-d\cdot i\right)$$

Where *Y*(*t*) represents the *t*-th element in the output sequence, ∗_*d*_ denotes the convolution operator with dilation factors *d*, and *w*(*i*) is the weights of convolution kernel *w*.

**Fig. 3:**
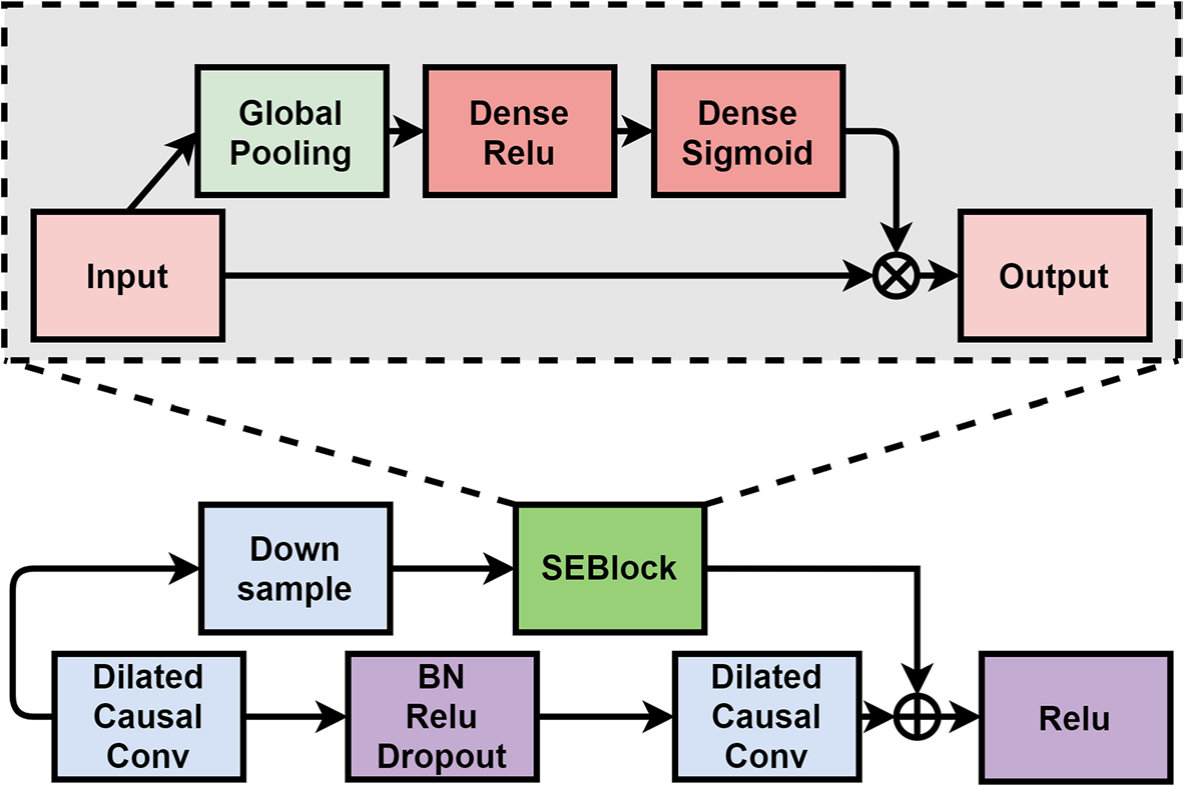
The internal structure of SE-TCNBlock

As shown in Fig. 3, we use the residual connection [37] in SE-TCNBlock to effectively train the deep neural network, which alleviates the gradient disappearance problem to some extent. Each SE-TCNBlock contains two channels, where the main channel of the residual connection contains two dilated causal convolution layers, and each convolution layer is activated after using batch normalization [38] and a rectified linear unit (ReLU) [39]. The dropout rate is set to 0.1, the dilated convolution factor d in the SE-TCNBlock is equal to 2^*L*^, where *L* = (1, 2, 3, 4, 5), and the RFS of the network is exponentially related to the number of layers, which is computed as follows:

4 $$\textrm{RFS}=1+2\cdot \left(k-1\right)\cdot \sum \limits_{i=1}^5{d}_i$$

Therefore, we enhance the RFS of the network by choosing a larger convolution kernel size *k*, increasing the dilation factor *d* or the number of the network layers *L*.

The sub-channel of the residual connection includes a downsampled convolutional layer with a convolutional kernel size of 1 (1 × 1 Conv) and an SEBlock.

SEBlock is a channel-wise attention mechanism module within SENet [40], that aims to capture the interdependencies of each channel in the feature map.

To capture dependencies between different lengths and time steps, this network employs varying dilation factors within each SE-TCNBlock. The blocks are hierarchically connected, with the output of each block feeding into the input of the next. The final output of the last SE-TCNBlock is the signal latent representation tensor denoted as *Z*_s_, which represents the feature-extracted representation of the original signal.

### Cross-modal feature fusion

The ordinary feature fusion approach can be divided into two types: early fusion and late fusion, depending on where the fusion occurs. Early Fusion or Feature-level Fusion, combines features from different modalities at the input level to obtain a richer representation. Late Fusion or Decision-level Fusion, involves using different models to extract features from different modalities and then integrating the prediction results of these models at the decision level.

Both early fusion and late fusion have their advantages and limitations. Early fusion can provide a holistic representation of information from different modalities but may not effectively capture the relationships between features. Late fusion, on the other hand, can model the relationships between features more flexibly but may require more computational resources and time.

**Fig. 4:**
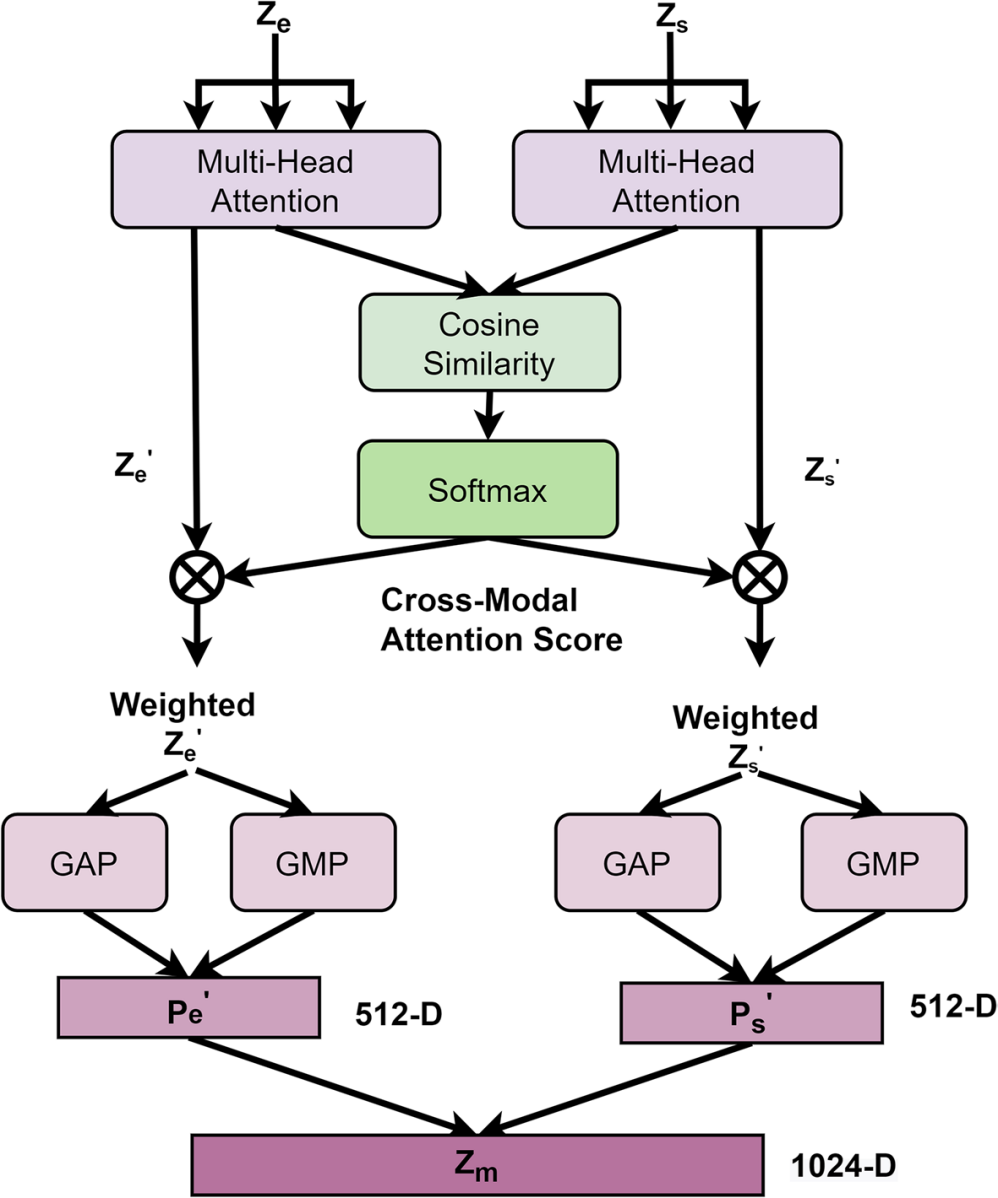
The internal structure of CMFF

In the CMFF module presented in Fig. 4, a multi-head attention mechanism [41] is utilized to measure the similarity between the latent representation tensors of the signal (denoted as *Z*_s_) and the expert (denoted as *Z*_*e*_). The purpose of this module is to fuse the features from different modalities and capture the cross-modal interactions for improved performance in the given task.

In the multi-head attention mechanism, each representation tensor is linearly projected to a set of vectors with different semantics, denoted as ${Q}_{\textrm{i}}={Z}_{\textrm{i}}\ast {W}^{Q_{\textrm{i}}}$, ${K}_{\textrm{i}}={Z}_{\textrm{i}}\ast {W}^{K_{\textrm{i}}}$, and ${V}_{\textrm{i}}={Z}_{\textrm{i}}\ast {W}^{V_{\textrm{i}}}$. where i ∈ {e, s}, ${W}^{Q_{\textrm{i}}}$, ${W}^{K_{\textrm{i}}}$, and ${W}^{V_{\textrm{i}}}$ denote the query matrix, key matrix and value matrix respectively. Then, these vectors are divided into 8 attention heads, and each head performs self-attention calculation independently. The weight matrices of each head are then concatenated together. Finally, the output tensor of the multi-headed attention mechanism is computed as follows:

5 $$MultiHeadAttention\left({Q}_{\textrm{i}},{K}_{\textrm{i}},{V}_{\textrm{i}}\right)= Concat\left({head_{\textrm{i}}}^1,\dots, {head_{\textrm{i}}}^8\kern0em \right){W}^{O_{\textrm{i}}},\textrm{i}\in \left\{\textrm{e},\textrm{s}\right\}$$

6 $${head_{\textrm{i}}}^n= softmax\left(\frac{{Q_{\textrm{i}}}^n{\left({K_{\textrm{i}}}^n\right)}^T}{\sqrt{d_{model}}}\right){V_i}^n,\textrm{i}\in \left\{\textrm{e},\textrm{s}\right\},{d}_{model}=32$$

7 $$softmax\left({x}_i\right)=\frac{\exp \left({x}_i\right)}{\sum \limits_{j=1}^n\exp \left({x}_j\right)}$$

where ${W}^{O_{\textrm{i}}}$ denotes the output weight matrix. In *head*_i_^*n*^, the superscript *n* belongs to the set {1, 2, …, 8} and indicates the number of attention heads, the subscript i belongs to the set {e, s}.

We denote the outputs of *Z*_e_ and *Z*_s_ after multi-headed self-attention (intra-modal) as *Z*_e_^'^ and *Z*_s_^'^ respectively. We then calculate the cosine similarity between *Z*_e_^'^ and *Z*_s_^'^, and normalize them using the softmax function to obtain the cross-modal attention score (CMAS, inter-modal). Next, we weight the output of the multi-headed self-attention with the CMAS to obtain a weighted representation denoted as *Weighted* _ *Z*_i_^'^, i ∈ {e, s}. We also apply global average pooling (GAP) and global maximum pooling (GMP) on the weighted representation *Weighted* _ *Z*_i_^'^, and concatenate the resulting vectors to obtain a 512-dimensional tensor denoted as *P*_i_ ∈ ℝ^*B* ∗512^, i ∈ {e, s}. Finally, we concatenate *P*_e_ and *P*_s_, resulting in a multimodal fusion latent representation tensor denoted as *Z*_m_ ∈ ℝ^*B* ∗1024^. This fusion tensor contains information from both *Z*_e_ and *Z*_s_, combined through the CMAS and the pooling operations, which can be further used for downstream tasks or analyses.

*Z*_m_(m is an abbreviation for multimodal) is calculated as follows:

8 $$cosine\_ similarity\left({Z_{\textrm{e}}}^{\hbox{'}},{Z_{\textrm{s}}}^{\hbox{'}}\right)=\frac{{Z_{\textrm{e}}}^{\hbox{'}}\cdot {Z_{\textrm{s}}}^{\hbox{'}}}{\left|{Z_{\textrm{e}}}^{\hbox{'}}\right|\left|{Z_{\textrm{s}}}^{\hbox{'}}\right|}$$

9 $$CMAS= softmax\left( cosine\_ similarity\left({Z_{\textrm{e}}}^{\hbox{'}},{Z_{\textrm{s}}}^{\hbox{'}}\right)\right)$$

10 $$Weighted\_{Z_{\textrm{i}}}^{\hbox{'}}= CMAS\cdot {Z_{\textrm{i}}}^{\hbox{'}},\textrm{i}\in \left\{\textrm{e},\textrm{s}\right\}$$

11 $${P}_{\textrm{i}}= concat\left( GAP\left( Weighted\_{Z_{\textrm{i}}}^{\hbox{'}}\right), GMP\left( Weighted\_{Z_{\textrm{i}}}^{\hbox{'}}\right)\right),\textrm{i}\in \left\{\textrm{e},\textrm{s}\right\}$$

12 $${Z}_{\textrm{m}}= concat\left({P}_{\textrm{e}},{P}_{\textrm{s}}\right)$$

Overall, the CMFF module combines the strengths of different modalities and captures their complementary information, which can improve the performance of subsequent classification tasks (as discussed in Experiment three).

## Results

In this section, we conducted three main experiments. Firstly, we performed hyperparameter analysis by tuning the hyperparameters of the model to study their impact on the experimental results. Secondly, we compared different signal backbone models to investigate their performance differences in the cross-modal feature fusion task. Finally, we conducted ablation experiments by comparing the performance of single-modal and multi-modal inputs to validate the effectiveness of the CMFF method. The results confirmed that our proposed model achieved the best accuracy (96.8%).

### Experimental setup

#### Dataset

**Fig. 5:**
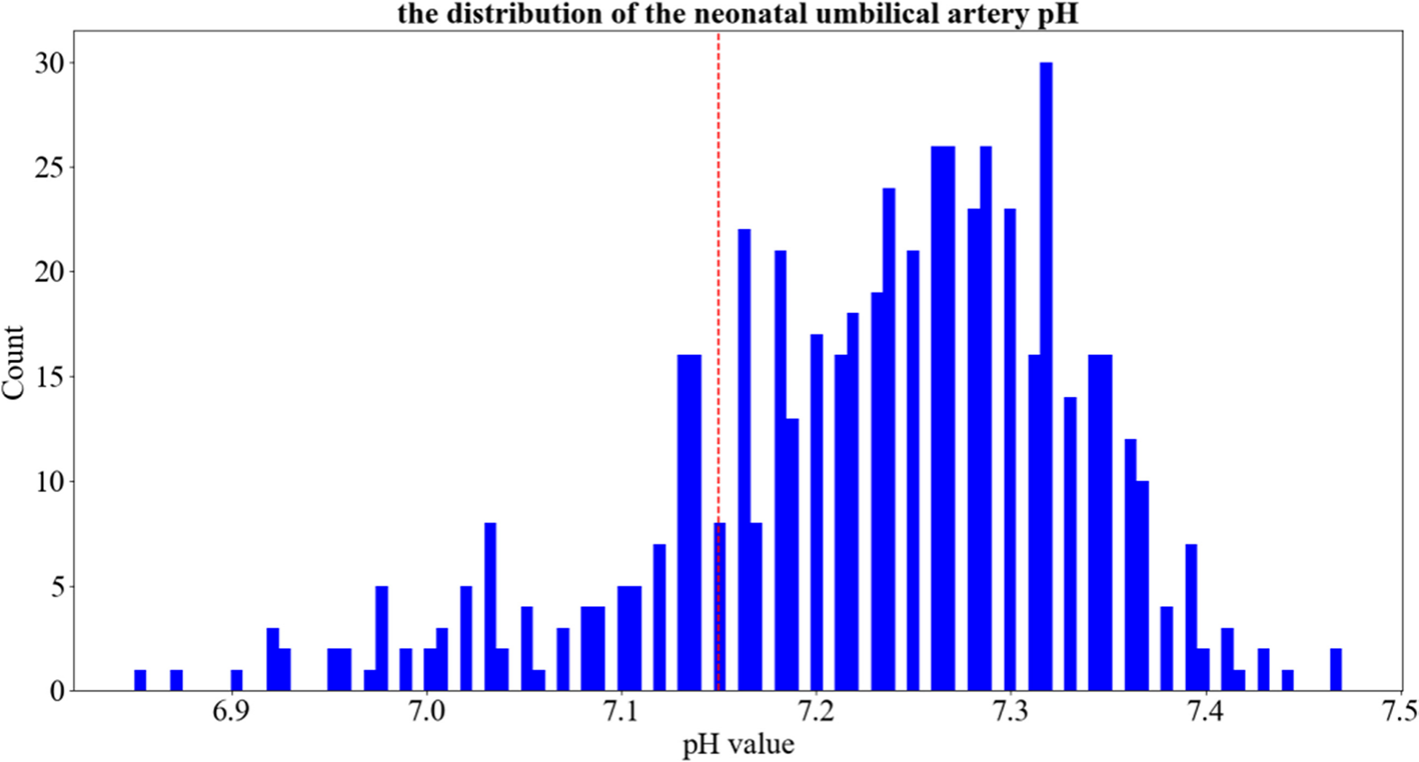
The distribution of the neonatal umbilical artery pH

The data used in this study were obtained from CTU-UHB [42, 43], a database of CTG recordings, containing a total of 552 samples with a sampling frequency of 4 Hz. Each CTG recording contains a set of FHR signals and a set of UC signals. In order to accurately assess intrauterine fetal acidosis, it is crucial to integrate these signals with clinical indicators. One such indicator is the neonatal umbilical artery pH, which serves as one of the gold standards for evaluating the presence of acidosis in the intrauterine environment. The lower the pH value, the more severe the fetal hypoxia. Different clinical doctors or research institutions may use different pH thresholds to determine whether the fetus is hypoxic, depending on their clinical experience and actual situation. We referred to the most commonly used criteria for delineation at this stage [8, 13, 14, 16, 26] and used 7.15 as a threshold value, with a pH value below 7.15 considered pathological and one greater than or equal to 7.15 considered normal, yielding a total of 447 normal and 105 pathological records. The distribution of pH values in the umbilical artery of newborns in the dataset is shown in Fig. 5.

#### Data preprocessing

**Fig. 6:**
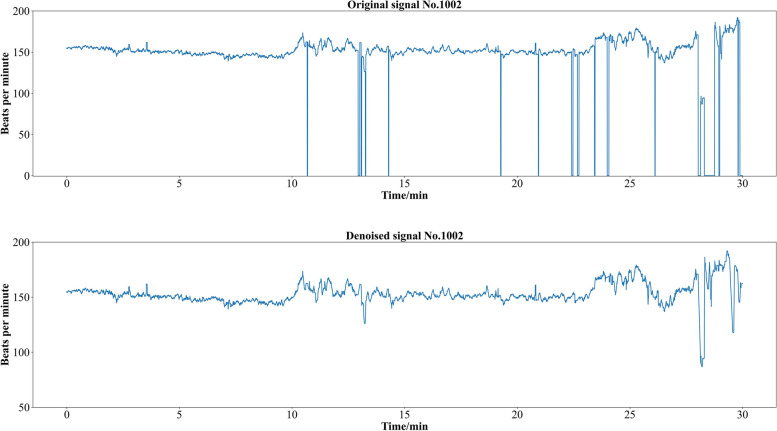
Comparison of original signal (Top) and denoised (Bottom) signal. Outliers and missing values are removed from FHR signals using a mini-batch-based minimized sparse dictionary learning approach

Noises during recording may disrupt the FHR signal, compromising its quality and impacting diagnostic tasks. Additionally, the imbalance between positive and negative samples poses a challenge, requiring data augmentation to increase the number of pathological samples. To overcome the challenges mentioned above, we adopted the preprocessing and data augmentation methods previously proposed by our group [44, 45] to enhance the original signal, and the denoised signal is shown in Fig. 6. Firstly, to ensure high integrity and quality of the signals used, signals of effective lengths below 10,000 (severely incomplete) were discarded and a total of 524 samples (pathological: 95, normal: 429) were used. Secondly, noise disturbances such as missing values are removed using a mini-batch-based minimized sparse dictionary learning approach [43], and all 524 records had an effective length greater than or equal to 10,000. Thirdly, since fetal distress mainly occurs before delivery, we focused on the last 30 minutes of each sample in our experiments, which corresponds to a sample length of 7200 (4 Hz). Finally, the pathological FHR signals were synthesized using a Generative Adversarial Network (GAN) [45] to make the sample distribution balanced. GAN is used only for the training set, and the information in the test set is not used to synthesize data samples; therefore, the evaluation process is reliable and generalized.

#### Evaluation

We first randomly sampled 444 samples (pathological: 55, normal: 389) from the original 524 samples for training, and used 80 samples (pathological: 40, normal: 40) for testing. Next, we used GAN for data augmentation on the training set to balance the positive and negative samples (pathological: 389, normal: 389). During the training process, we further divided the training set into training and validation sets using 5-fold cross-validation, and evaluated the model on the original test set. The average of the predictions from 5-fold cross-validation was used as the final prediction result. We calculated several metrics including accuracy (Acc), precision (Pre), sensitivity (Sen), specificity (Spe), and F1-Score (F1).

### Experiment one: Hyperparameter optimization

**Fig. 7:**
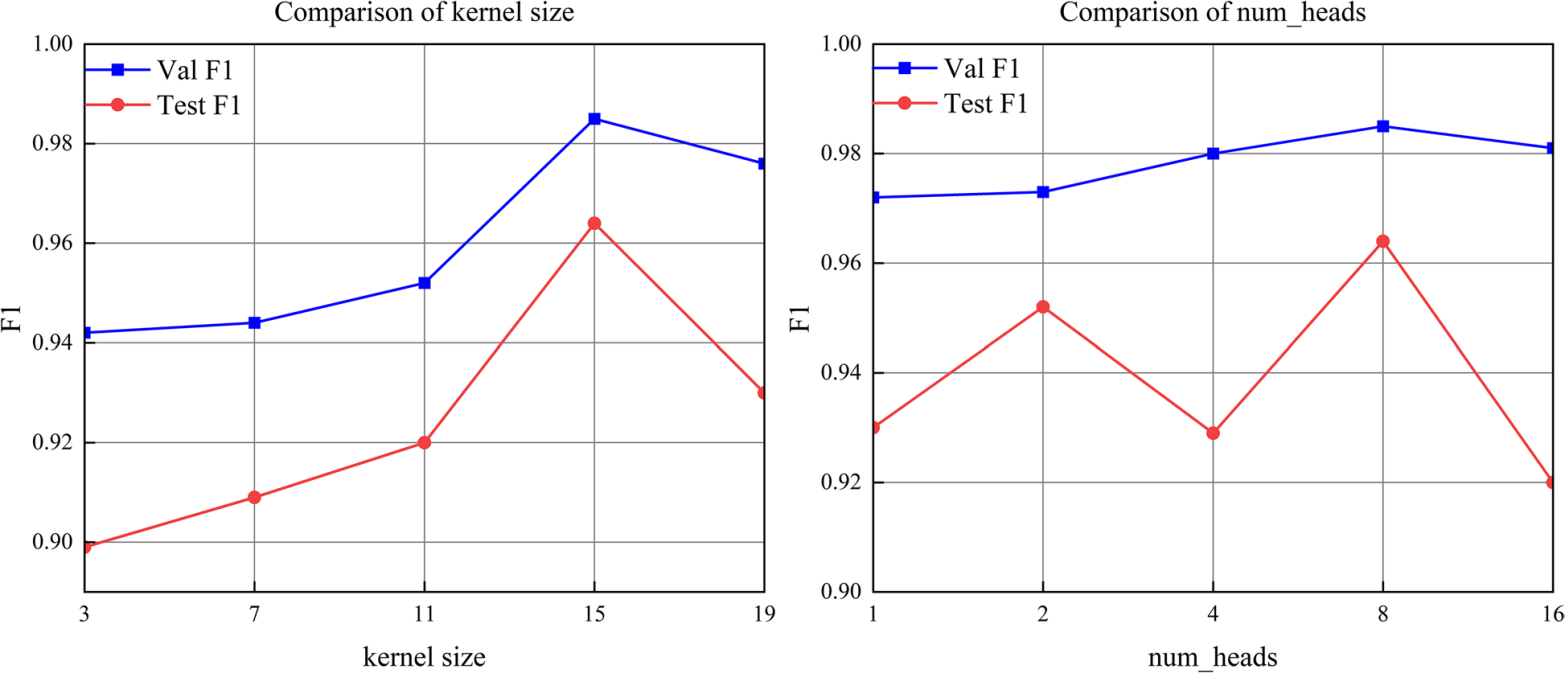
Effect of different kernel sizes (left) and num_heads (right) on the model

To achieve optimal model performance, we conducted a thorough analysis of different hyperparameter settings and their impact on classification results. Our experimental findings revealed that the kernel size in the SE-TCNBlock and the number of heads in the multi-headed attention mechanism significantly influenced the classification performance, as illustrated in Fig. 7. The remaining hyperparameters were set to their default values, as follows: the cross-entropy loss function and the Adam optimizer [46] were utilized in the training process. The batch size was set to 16, and the training duration was configured for 120 epochs, with early stopping [47]. The learning rate strategy employed cosine annealing with an initial learning rate of 2.5e-4 and a decay factor set to 0.8.

When the kernel size was increased from 3 to 15, a remarkable improvement in F1 scores was observed for both the validation set and the test set, indicating superior performance. However, when the kernel size was further increased to 19, a slight drop in the F1 score for the validation set and a more significant drop to 0.93 for the test set were observed, implying that larger kernel sizes may not always yield better results. Similarly, increasing num_heads from 4 to 8 resulted in a successive improvement in F1 scores for both the validation and test sets, suggesting that incorporating more attentional heads can enhance the model’s performance. Nevertheless, when num_heads continued to increase to 16, a slight decrease in the F1 score for the validation set and a more substantial drop to 0.92 for the test set were observed. This suggests that excessively large num_heads may lead to over-complexity and overfitting, ultimately negatively impacting the model’s performance.

In summary, the experimental results suggest that a moderate kernel size and num_heads may help to improve the performance of the model, but too large a kernel size and num_heads may have a negative impact on performance. Therefore, in this paper we set the kernel size and num_heads to 15 and 8 respectively.

### Experiment two: comparing different signal backbone

In order to substantiate the superiority of the SE-TCN model, Experiment two involved a meticulous comparison of various Signal Backbone models, including ResNet18, ResNext18, Inception, VGG16, SE-ResNet18, and SE-ResNext18. Notably, we exclusively replaced the signal backbone component while keeping the expert feature module and cross-modal feature fusion module unchanged. Moreover, consistent datasets were employed for training and testing, and identical hyperparameter settings, were utilized to ensure utmost fairness and reliability of the experiments.

**Fig. 8:**
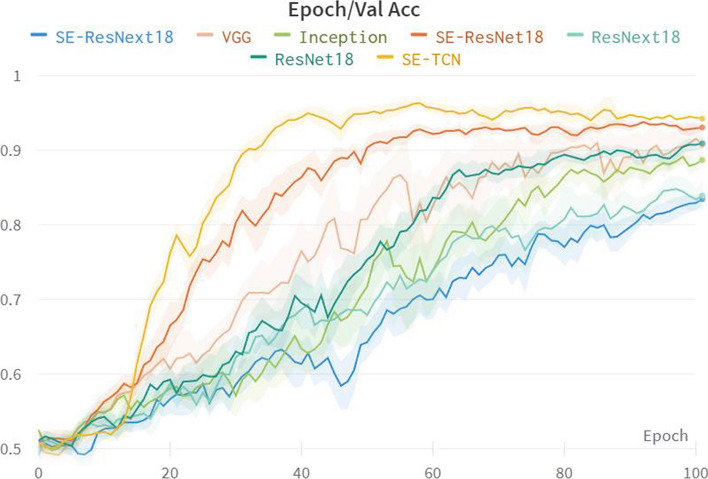
The comparison results of the Accuracy of different Signal Backbone models on the validation set

**Fig. 9:**
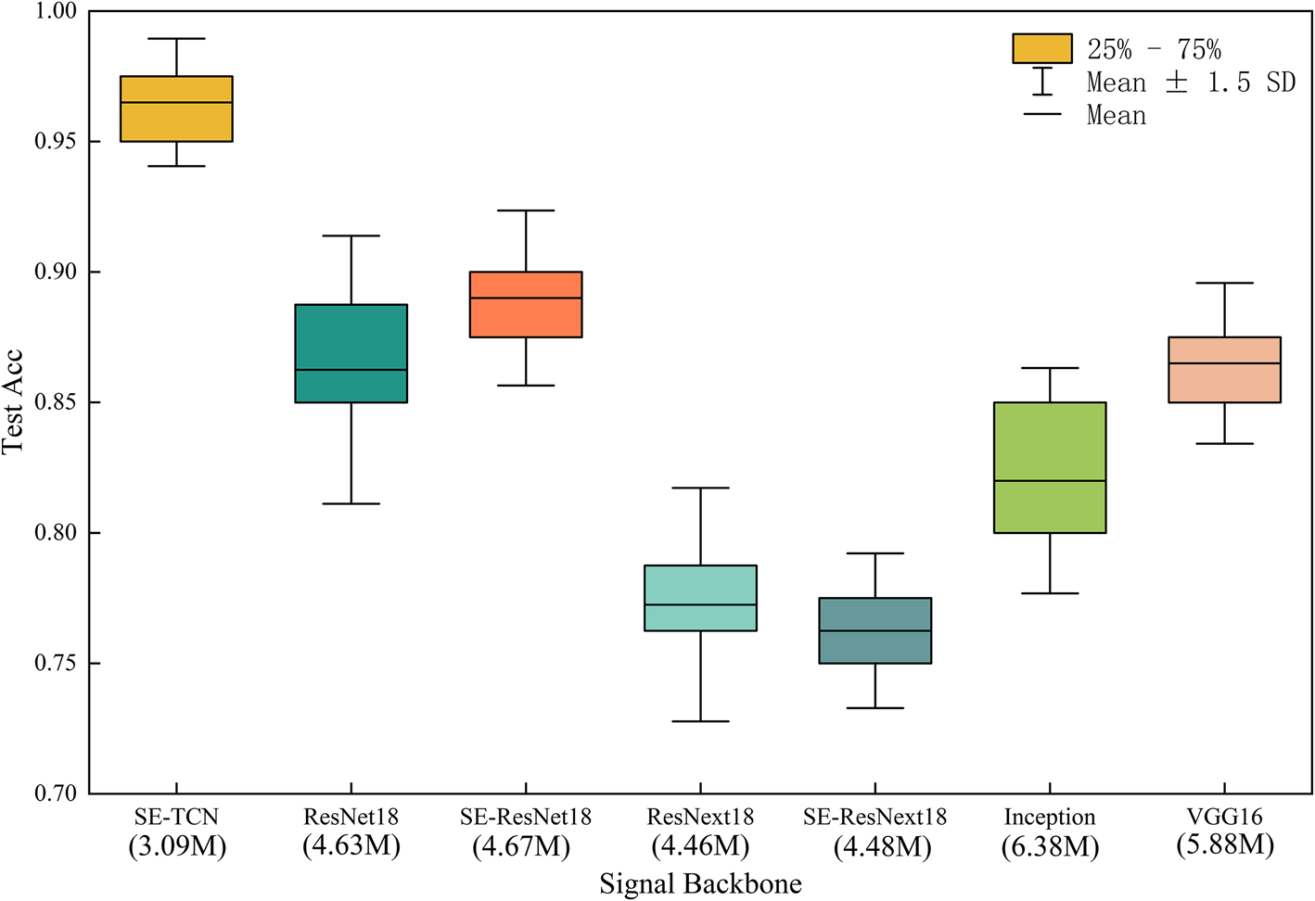
The boxplots of the accuracy of different Signal Backbone models on the test set. The numbers in brackets on the x-axis indicate the total number of parameters for each signal backbone model. SD stands for standard deviation

Figure 8 represents the average accuracy curves of different signal backbone models on the validation set during the training process, while Fig. 9 depicts the average accuracy of different signal backbone models on the test set. The experimental results clearly demonstrate that the SE-TCN model exhibits a significant advantage, achieving an average accuracy of 0.968 on the test set, compared to the accuracy range of 0.7725 to 0.89 for other models. Notably, the SE-TCN model surpasses the SE-ResNet18 and SE-ResNext18 models by 7.5 and 20.2% in terms of accuracy, respectively. This indicates that the SE-TCN model excels in feature extraction and cross-modal fusion, resulting in a noteworthy improvement in model accuracy. Furthermore, the SE-TCN model boasts a smaller total number of parameters, totaling at 3.09 M, which makes it more lightweight compared to other models.

In summary, the SE-TCN model holds promising potential for applications in multi-modal signal processing tasks, as it demonstrates high accuracy while minimizing the number of parameters, making it a favorable choice for a high-performance and low-complexity model.

### Experiment three: ablation experiments

In Experiment three, we conducted ablation experiments to thoroughly investigate the effects of different components in the Hybrid-FHR architecture. Specifically, we compared the performance of (1) using only expert features, (2) using only the signal backbone model (SE-TCN), and (3) using the complete Hybrid-FHR architecture. Furthermore, to demonstrate the importance of the proposed CMFF module, we compared early and late fusion approaches. In early fusion, the expert latent representation tensor and signal latent representation tensor are fused through simple concatenation. In late fusion, the two different modality tensors are each passed through their respective classification heads and then fused with a 1:1 decision weight.

According to the results of ablation experiments (Table 2), when considering only a single type of expert features, the order of the three expert feature types is: frequency domain > morphological time domain > nonlinear. The performance of single-modal features decreased to a certain extent compared to using the complete Hybrid-FHR architecture. When using all expert features, the accuracy decreased by 10 to 86.8% compared to the complete architecture, and when using signal features, the accuracy decreased by 4.8 to 92.0%. This indicates that the fusion of multimodal information is of great significance for improving the diagnostic accuracy and efficiency in medical diagnosis. Furthermore, in the comparison of different fusion methods, the late fusion performed slightly better than the early fusion, but still lower than our proposed CMFF method. This indicates that the CMFF method can better fuse different modal information and improve the classification performance of the model.

**Table 2:** Performance comparison of different modal features and different fusion methods

Method	Acc%	Spe%	Sen%	Pre%	F1%
Morphological time domain features only	79.3	76	82.5	77.5	79.9
Frequency domain features only	80.8	77.5	84	78.9	81.4
Nonlinear features only	75.5	80.5	70.5	78.3	74.2
All expert features	86.8	83.5	90.0	84.5	87.1
Signal features only	92.0	95.0	89.0	94.7	91.8
Early fusion (concatenation fusion)	93.5	97.0	90.0	96.8	93.3
Late fusion (decision-level fusion)	95.8	96.0	95.5	96.0	95.7
Hybrid-FHR (Expert features + Signal features + CMFF)	**96.8**	**97.5**	**96.0**	**97.5**	**96.7**

In Table 3, we compared the generalization error of the model in two scenarios: with and without expert features. we can see that the generalization error of the model is reduced from 4.9 to 3% after incorporating the expert features. This indicates that incorporating expert features helps to reduce the generalization error of the model and prevents the risk of overfitting.

**Table 3:** Comparison of generalization error with and without expert features

Method	Train Acc%	Test Acc%	Generalization error%
Without expert features	96.9	92.0	4.9
With expert features	99.8	96.8	3

**Fig. 10:**
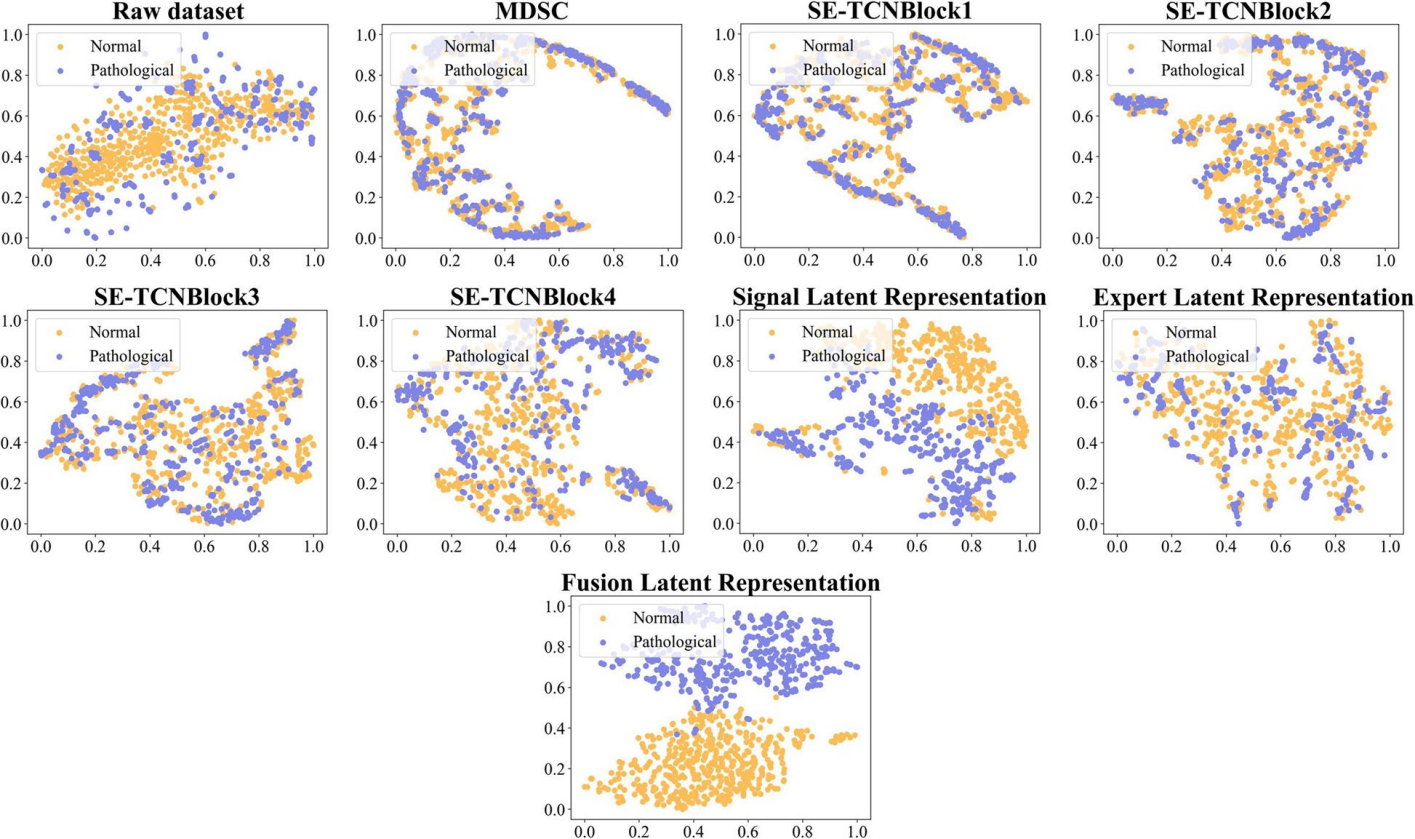
The Visualization Output of each Layer in the Hybrid-FHR using t-SNE

We plotted a t-distribution stochastic neighbor embedding (t-SNE) to visualize the output of each layer, as shown in Fig. 10. Initially, the raw data distribution appears scattered and lacks clear decision boundaries. However, as the network undergoes successive layers of feature extraction, the t-SNE plot gradually reveals distinct and separable clusters. This suggests that the network progressively learns and captures informative representations, leading to more discriminative features. Remarkably, the fusion latent representation output by CMFF forms visually distinct and well-separated clusters in the t-SNE plot. These evident clusters showcase the ability of CMFF to accurately capture and differentiate underlying patterns within the data.

## Discussion

Table 4 offers a comprehensive overview of the various approaches proposed by researchers over the last few decades for fetal acidosis diagnosis. As show in Table 4 Most of the existing studies use only single modal features (e.g., expert features, or 1D signal features).

**Table 4:** Comparison of different algorithms for fetal acidosis diagnosis

Reference	Method	Database	Division criterion	Performance%
[1]	Expert +1D-CNN	CTU-UHB	pH < 7.15	Sen: 75.23; Spe: 70.82
[5]	Expert + PCA + ANN	Private	pH < 7.1	Sen:60.3; Spe:67.5
[6]	Expert + RELIEF + AdaBoost	CTU-UHB	pH < 7.05	Sen:64.1; Spe:65.2
[7]	Expert + Random Forest	CTU-UHB	Expert annotation	Sen:72.41; Spe:78.4
[16]	1D-CNN-GRU	CTU-UHB	pH < 7.15	Acc: 95.15; Sen: 96.20 Spe: 94.09; Pre: 94.21
[26]	Expert + SVM / Naive Bayes	CTU-UHB	pH < 7.15	Sen: 73.4 / 72.3; Spe: 76.3 / 75.6
Current work	Hybrid-FHR (Expert + SE-TCN + CMFF)	CTU-UHB	pH < 7.15	Acc: **96.8;** Sen: **96.0** Spe: **97.5;** Pre: **97.5** F1: **96.7**

*AdaBoost* Adaptive Boosting, *GRU* gated recurrent units, *SVM* support vector machine

To ensure fairness, we only compared algorithms [1, 16, 26] that utilized the CTU-UHB dataset and employed a pH threshold of 7.15 as the division criterion. We can draw several conclusions from Table 4. Firstly, our algorithm outperforms the state-of-the-art algorithms reported in previous literature, achieving the best performance on five different metrics. Secondly, comparing [16, 26], algorithms based on 1D signal features perform much better than algorithms based on expert features, demonstrating the advantage of DL over traditional ML methods. Thirdly, we notice some similarities between our approach and [1], which also incorporates expert features that are fused with 1D signal features. Nevertheless, it is worth noting that [1] employs a simple 1D-CNN model for extracting 1D signal features, followed by a late fusion at the decision level. In contrast, we utilized the SE-TCN as backbone network, which boasts superior long sequence signal feature extraction capabilities compared to conventional CNNs. Additionally, we introduced the CMFF module at the feature level, which explicitly models the correlation and difference between different modalities and further improves the classification effect.

In this work, we present an intelligent analysis algorithm Hybrid-FHR for diagnosing fetal acidosis. This algorithm can be integrated into clinical practice to aid obstetricians in making accurate medical decisions by considering the extracted expert feature parameters and the final predicted probability results. Based on the experimental results, we draw the following conclusions: (a.) Multimodal features lead to better classification results than using signal features or expert features alone. (b.) SE-TCN can effectively extract complex features from FHR signals, and outperforms six different baseline models in terms of convergence speed and parameter size. (c.) Both late fusion and early fusion methods achieve satisfactory results, but they are still inferior to our proposed CMFF method in terms of accuracy.

Our algorithm in obstetrics and perinatal care holds significant practical implications by providing accurate and timely assessments of fetal distress. It facilitates early identification, leading to timely clinical interventions and preventing complications for both the mother and fetus. The algorithm reduces the diagnostic burden on healthcare professionals, automating aspects of diagnosis and allowing them to focus on critical patient care. Additionally, its computational nature makes it suitable for telemedicine applications, enabling remote monitoring and diagnosis, especially in areas with limited access to specialized healthcare facilities. In conclusion, our fetal distress diagnosis algorithm has the potential to enhance diagnostic efficiency, accuracy, and timeliness, positively impacting patient outcomes and overall perinatal care quality.

## Conclusions

In this study, we propose a novel artificial intelligence algorithm called Hybrid-FHR for fetal acidosis diagnosis using multimodal features of the FHR signal. Our algorithm consists of three key components. First, we designed the SE-TCN backbone network to extract one-dimensional spatiotemporal representations from the FHR signal. Second, we incorporated three types of expert features including morphological time domain, frequency domain, and nonlinear parameters based on expert prior knowledge. Finally, we developed a cross-modal feature fusion (CMFF) method, which employs a multi-headed attention mechanism for fusing signal representations with expert feature representations.

We evaluate our algorithm against six baseline models and two fusion approaches on a publicly available dataset of FHR recordings. Our results demonstrate that Hybrid-FHR outperforms the existing methods in terms of accuracy (96.8%) and efficiency. With the increasing number of publicly available datasets, we will apply the algorithm proposed in this study to different datasets to increase the robustness and generalizability of the model, while considering interpretable analysis to help clinicians make more objective and accurate decisions.

### Supplementary Information

**Additional file 1.**

**Additional file 2.**

**Additional file 3.**

## Data Availability

The CTU-UHB database is a publicly available resource (https://physionet.org/content/ctu-uhb-ctgdb/1.0.0/).

## Abbreviations

1D-CNN: One-dimensional convolutional neural network
Acc: Accuracy
ANN: Artificial neural network
ANS: Autonomic nervous system
CMAS: Cross-modal attention score
CMFF: Cross-modal feature fusion
CWT: Continue wavelet transform
CTG: Cardiotocography
EFM: Electronic fetal monitoring
F1: F1-Score
FIGO: the International federation of gynecology and obstetrics
FFT: Fast fourier transform
FHR: Fetal heart rate
FHRV: Fetal heart rate variability
FetMov: Fetal movement
GAN: Generative adversarial network
GAP: Global average pooling
GLCM: Gray level co-occurrence matrix
GMP: Global maximum pooling
GRU: Gate recurrent unit
MDSC: Multi-scale depthwise separable convolution
PCA: Principal component analysis
Pre: Precision
RBF-SVM: Radial basis function support vector machine
RELIEF: RELevance in estimating features
ReLU: Rectified linear unit
RFE: Recursive feature elimination
SE-TCN: Squeeze and excitation temporal convolutional network
Sen: Sensitivity
Spe: Specificity
ST: Statistical testing
STFT: Short-time fourier transform
t-SNE: t-distribution Stochastic neighbor embedding
UC: Uterine contraction
