# Supplementary material for: Hybrid-FHR: a multi-modal AI approach for automated fetal acidosis diagnosis
Source: BMC Med Inform Decis Mak. 2024 Jan 22;24:19. doi: 10.1186/s12911-024-02423-4 (PMC10801938; doi:10.1186/s12911-024-02423-4)
Supplement: Supplementary file 1 — Additional file 1. [file 12911_2024_2423_MOESM1_ESM.docx]

Additional file 1. Description of morphological time domain features extracted in this paper

| Feature name | Description or formula |
| --- | --- |
| Baseline (BL) [19] | The average value of the FHR signal after removing the acceleration and deceleration modes. |
| nAcc [19] | The number of times that the amplitude increases beyond the baseline level by 15 bpm and the duration exceeds 15 s but is less than 10 min. |
| nDec [19] | The number of times that the FHR was reduced in amplitude by more than 15 bpm above the baseline level for a duration of more than 15 s. |
| max_rr | $max(RR)$ |
| min_rr | $min(RR)$ |
| mean_rr | $mean(RR)$ |
| median_rr | $median(RR)$ |
| std_rr | $\sqrt{\frac{1}{n}\sum_{i=1}^{n} (RR_{i}-\text{mean\_rr})^{2}}$  The standard deviation of the RR interval sequences, where $n$ is the length of the RR interval sequences. |
| skew_rr | $\frac{\sqrt{\frac{1}{n}\sum_{i=1}^{n} (RR_{i}-\text{mean\_rr})^{3}}}{\text{std\_rr}^{3}}$  The skewness of the RR interval sequences. |
| kurt_rr | $\frac{\sqrt{\frac{1}{n}\sum_{i=1}^{n} (RR_{i}-\text{mean\_rr})^{4}}}{\text{std\_rr}^{4}}$  The kurtosis of the RR interval sequences. |
| SDNN | $\sqrt{\frac{1}{n-1}\sum_{i=1}^{n-1} (NN_{i}-\text{mean}(NN))^{2}}$  The standard deviation of NN. |
| RMSSD | $\sqrt{\text{mean}(NN^{2})}$  The root mean square standard deviation of NN. |
| NN50 | Number of NN sequences with absolute values greater than 50ms. |
| pNN50 | NN50 as a percentage of the total number of heartbeats. |
| STV [20] | $\frac{\sum_{i=1}^{24M} \left\vert sm(i+1)-sm(i)\right\vert}{24M}$  STV is computed according to the average values of 2.5-s blocks in the FHR signal. $sm(i)$is used to show the i-th 2.5s block of the signal, so there are 24 $sm(i)$ in a minute. M shows the number of minutes of the FHR. |
| LTV [20] | $\frac{1}{M}\sum_{i=1}^{M} \left[\max_{i\in M} (x(i))-\min_{i\in M} (x(i))\right]$  The FHR signal is first divided into 60s blocks. These blocks are symbolized with $x(i)$ as above. As mentioned above, M stands for the number of minutes of the FHR. |
| Tri [21] | The HRV triangular index measurement is the integral of the density distribution (= the number of all NN-intervals) divided by the maximum of the density distribution. |
| TINN [22] | The triangular interpolation of the NN interval histogram |
