# Supplementary material for: Hybrid-FHR: a multi-modal AI approach for automated fetal acidosis diagnosis
Source: BMC Med Inform Decis Mak. 2024 Jan 22;24:19. doi: 10.1186/s12911-024-02423-4 (PMC10801938; doi:10.1186/s12911-024-02423-4)
Supplement: Supplementary file 2 — Additional file 2. [file 12911_2024_2423_MOESM2_ESM.docx]

Additional file 2. Description of frequency domain features extracted in this paper

| Feature name | Description or formula |
| --- | --- |
| rr_VLF | $\left\vert X(f)_{0-0.03}\right\vert^{2}=\left\vert\int_{0}^{0.03} x(t)e^{-2\pi ift}dt\right\vert^{2}$  Power spectral density in the VLF band, where $x(t)$denotes the time domain signal,$X(f)$is the frequency domain signal of x(t) after Fourier transformation and$i$represents the imaginary unit. |
| rr_LF | $\left\vert X(f)_{0.03-0.05}\right\vert^{2}=\left\vert\int_{0.03}^{0.05} x(t)e^{-2\pi ift}dt\right\vert^{2}$  Power spectral density in the LF band. |
| rr_MF | $\left\vert X(f)_{0.05-0.15}\right\vert^{2}=\left\vert\int_{0.05}^{0.15} x(t)e^{-2\pi ift}dt\right\vert^{2}$  Power spectral density in the MF band. |
| rr_HF | $\left\vert X(f)_{0.05-1}\right\vert^{2}=\left\vert\int_{0.05}^{1} x(t)e^{-2\pi ift}dt\right\vert^{2}$  Power spectral density in the HF band. |
| rr_Total_Power | $\left\vert X(f)_{0-1}\right\vert^{2}=\left\vert\int_{0}^{1} x(t)e^{-2\pi ift}dt\right\vert^{2}$  Total power spectral density. |
| rr_percent_VLF | $\frac{\text{rr\_VLF}}{\text{rr\_Total\_Power}}$  Percentage of VLF in the total energy band. |
| rr_percent_LF | $\frac{\text{rr\_LF}}{\text{rr\_Total\_Power}}$  Percentage of LF in the total energy band. |
| rr_percent_MF | $\frac{\text{rr\_MF}}{\text{rr\_Total\_Power}}$  Percentage of MF in the total energy band. |
| rr_percent_HF | $\frac{\text{rr\_HF}}{\text{rr\_Total\_Power}}$  Percentage of HF in the total energy band. |
| rr_ratio | $\frac{\text{rr\_LF}}{\text{rr\_MF}+\text{rr\_HF}}$  Ratio of low frequency energy to medium frequency and high frequency. energy. |
| rr_peakVLF | Peak frequency in the VLF band. |
| rr_peakLF | Peak frequency in the LF band. |
| rr_peakMF | Peak frequency in the MF band. |
| rr_peakHF | Peak frequency in the HF band. |
