# Supplementary material for: Hybrid-FHR: a multi-modal AI approach for automated fetal acidosis diagnosis
Source: BMC Med Inform Decis Mak. 2024 Jan 22;24:19. doi: 10.1186/s12911-024-02423-4 (PMC10801938; doi:10.1186/s12911-024-02423-4)
Supplement: Supplementary file 3 — Additional file 3. [file 12911_2024_2423_MOESM3_ESM.docx]

Additional file 3. Description of nonlinear features extracted in this paper

| Feature name | Description |
| --- | --- |
| SD1 [28] | $std(\frac{RR[:-1]-RR[1:]}{\sqrt{2}})$  Short-axis deviations of the Poincaré scatter plot. $RR[:-1]$represents the RR interval sequence with the last point removed, $RR[1:]$represents the RR interval sequence with the first point removed |
| SD2 [28] | $std(\frac{RR[-1]+RR[1:]}{\sqrt{2}})$  Long-axis deviations of the Poincaré scatter plot. |
| SD_Ration [28] | $\frac{\text{SD1}}{\text{SD2}}$  Ratio of short axis deviations to long axis deviations. |
| ApEn [29] | The ApEn value of the time-series $X=\{x(i),1\leq i\leq N\}$of$N$points can be calculated by: $\text{ApEn}(m,\tau,r)=\Phi^{(m)}(r)-\Phi^{(m+1)}(r)$ $\Phi^{(m)}(r)=\frac{1}{N-m\tau+1}\sum_{i=1}^{N-m\tau+1} \ln[C_{i}^{m}(r)]$ $C_{i}^{m}(r)=\frac{N_{i}^{m}(r)}{N-m\tau+1}$ $X_{m}(i)=\{x(i),x(i+\tau),...x(i+(m-1)\tau)\},1\leq i\leq N-m\tau+1$ $d_{i,j}=\max(\left\vert x(i+k)-x(j+k)\right\vert,0\leq k\leq m-1)$           Where$m$indicates the embedding dimension, $\tau$indicates the time delay, and$r$indicates the tolerance. For a given subsequences $X_{m}(i)$, $N_{i}^{m}(r)$indicates the number of subsequences $X_{m}(j)$ are within distance $r$of $X_{m}(i)$(i.e. $d_{i,j}\leq r$).  In this paper, we set $m=2$ , $\tau=1$ , and $r=0.2\cdot\text{std\_rr}$. |
| SampEn [29] | $\text{SampEn}(m,\tau,r)=-\ln\frac{C^{m+1}(r)}{C^{m}(r)}$ |
| FuzzyEn [29] | $\text{FuzzyEn}(m,\tau,r)=\ln\Phi^{(m)}(r)-\ln\Phi^{(m+1)}(r)$ $\Phi^{(m)}(r)=\frac{1}{N-m\tau+1}\sum_{i=1}^{N-m\tau+1} (\frac{1}{N-m\tau}\sum_{j=1,j\neq i}^{N-m\tau} D_{i,j})$ $D_{i,j}=e^{-\ln2(d_{i,j}/r)^{2}}$ $d_{i,j}=\max(\left\vert\left[x(i+k)-\overline{x_{i}}\right]-\left[x(j+k)-\overline{x_{j}}\right]\right\vert,0\leq k\leq m-1)$ $\overline{x_{i}}=\frac{1}{m}\sum_{k=0}^{m-1} x(i+k),\text{ }\overline{x_{j}}=\frac{1}{m}\sum_{k=0}^{m-1} x(j+k)$          Fuzzy entropy uses fuzzy membership function $D_{i,j}=e^{-(d_{i,j})^{n}/r}$ to measure the similarity of $X_{m}(i)$ and $X_{m}(j)$, $\overline{x_{i}}$and $\overline{x_{j}}$are the local mean of the vector $X_{m}(i)$ and $X_{m}(j)$. |
| ShannEn [30] | $\text{ShannEn = }-\sum_{x} P(x(i))\cdot\log_{2}P(x(i))$  Where$P(x(i))$denotes the probability of occurrence of $x(i)$. |
| LZC [31] | $s(i)=\left\{\begin{matrix} 1,\text{ if }x(i)>\text{threshold} \\ 0,\text{ otherwise} \end{matrix}\right.$ $C(N)=\frac{c(N)\cdot\log_{2}(N)}{N}$    First, the time series $X=\{x(i),1\leq i\leq N\}$of$N$points is converted into a binary sequence $S=\{s(i),1\leq i\leq N\}$ according to a threshold value, in this paper we use the mean value as the threshold. The second step is computing the distinct patterns in $S$, the complexity $c(N)$ is increased by 1 for each new pattern. Finally calculate the normalized $C(N)$. |
| FD [32] | Step1: Assume that the original sequence is $X=\{x(i),1\leq i\leq N\}$ and the length is $N$. For a range of $k$ values ranging from 1 to $k_{\max}$(in this paper, $k_{\max}=10$), construct $k$ new times series $X_{k}^{m}$ defined as follows: $X_{k}^{m}=\{x(m),x(m+k),x(m+2k),...,x(m+\left\lfloor\frac{N-m}{k}\right\rfloor k)\},m=\{1,2,...,k\}$   Step2: Calculate the length$L_{m}(k)$ of each curve $X_{k}^{m}$: $L_{m}(k)=\frac{\sum_{i=1}^{N-m/k} \left\vert x(m+ik)-x(m+(i-1)k)\right\vert(n-1)}{\left\lfloor\frac{N-m}{k}\right\rfloor k}$   Step3: Calculate the average value over $k$ sets of $L_{m}(k)$, for $m=\{1,2,...,k\}$, as $L(k)=\frac{1}{k}\sum_{m-1}^{k} L_{m}(k)$. Repeat the calculation for $k$ ranging from 1 to$k_{\max}$.  Step4: Calculate $\ln L(k)$and $\ln k$, and the slope is calculated using the linear fitting method and used as the value of the output fractal dimension FD:  $\text{FD}=polyfit(\ln(k),\ln(L(k)),1)$, where $polyfit(\ln(k),\ln(L(k)),1)$ indicates that the given data points are fitted using a one-time polynomial fit function. |
| Hurst [33] | Step1: The original sequence$X=\{x(i),1\leq i\leq N\}$of length $N$ is divided into $M$ subsequences of equal length $\frac{N}{M}$.  Step2: For each subsequences $Q_{m}=\{q(1),q(2),...,q(\frac{N}{M})\},1\leq m\leq M$, calculate its mean value$\overline{Q_{m}}$.  Step3: Calculate the deviation sequence$Y_{m}$for each subsequence: $Y_{m}=Q_{m}-\overline{Q_{m}}=\{y(1),...,y(\frac{N}{M})\}$   Step4: Calculate the cumulative deviation $Z_{m}$of each subsequence: $Z_{m}=\{Z_{m}(1),Z_{m}(2),...,Z_{m}(\frac{N}{M})\}=\{\sum_{i=1}^{1} y_{i},\sum_{i=1}^{2} y_{i},...,\sum_{i=1}^{N/M} y_{i}\}$   Step5: Calculate the maximum difference$R_{m}$ of the $Z_{m}$: $R_{m}=\max(Z_{1},Z_{2},...,Z_{m})-\min(Z_{1},Z_{2},...,Z_{m})$   Step6: Calculate the standard deviation $S_{m}$of each subsequence.  Step7: Calculate the RS value of each subsequence: $\text{RS}_{m}=\frac{R_{m}}{S_{m}}$   Step9: Calculate the average of RS values: $\text{ARS}_{M}=\sum_{m=1}^{M} \text{RS}_{m}$   Step10: Set different $M=\{M_{1},M_{2},...M_{n}\}$ and repeat step 1 - 9 to calculate multiple sets of $\text{ARS}=\{\text{ARS}_{M_{1}},\text{ARS}_{M_{2}},...\text{ARS}_{M_{n}}\}$.  Step11: Calculate Hurst using a one-time polynomial fit function: $\text{Hurst}=polyfit(\log10(\frac{N}{M}),\log_{10}\text{ARS},1)$  |
